# Bruxism Associated with Anoxic Encephalopathy: Successful Treatment with Baclofen

**DOI:** 10.1155/2013/129234

**Published:** 2013-12-17

**Authors:** A. Bruce Janati, Naif Saad ALGhasab, Fahad Saad ALGhassab

**Affiliations:** ^1^Center for Neurology of Fairfax, VA 22031, USA; ^2^King Faisal Specialist Hospital, Riyadh, Saudi Arabia; ^3^Hail University, 81442 Hail, Saudi Arabia

## Abstract

*Introduction*. Bruxism is a movement disorder characterized by grinding and clenching of the teeth. Etiology of bruxism can be divided into three groups: psychosocial factors, peripheral factors, and pathophysiological factors. *Methods*. The clinical investigation was conducted at King Khaled Hospital in Hail, Saudi Arabia, in 2012. *Results*. A 16-year-old Saudi female was brought to the hospital in a comatose state and with generalized convulsive seizures secondary to acute anoxic encephalopathy. In the third week of hospitalization, while still in a state of akinetic mutism, she developed incessant bruxism which responded favorably to a GABA receptor agonist (baclofen). *Conclusion*. Our data support the hypothesis that bruxism emanates from imbalance or dysregulation of the neurotransmitter system. Larger scale studies will be needed to confirm this hypothesis.

## 1. Introduction

The term “la bruxomanie” was first introduced by Marie Pietkiewicz in 1907 [1]. Bruxism is a diurnal or nocturnal parafunctional activity that includes tooth clenching, bracing, gnashing, and grinding. Its prevalence rates range from 5 to 96 percent in the adult population. Bruxism is of great interest to a wide variety of specialties. Although many etiologic factors such as stress and occlusal disorders have been proposed [2–5], bruxism's exact pathophysiology still remains unknown. Bruxism has been reported in certain neurological disorders such as Rett syndrome, mental retardation, anoxic encephalopathy, and cerebellar hemorrhage [6–8]. Tooth clenching, grinding, or both have been reported to be particularly prevalent in patients with idiopathic, tardive, and posttraumatic cranial dystonia, a neurological disorder manifested by abnormal spasms and movements involving the orolingual-facial musculature [9, 10]. Various treatment modalities have been reported to be useful for bruxism, but there is no general agreement as to what the best therapeutic option is. Most cases are treated with an occlusal appliance, while severe cases are treated with botulinum toxin injections [11]. We report for the first time successful treatment of bruxism with a gamma-aminobutyric acid (GABA) agonist.

## 2. Case Report

A 16-year-old Saudi female was brought to the emergency room of King Khaled Hospital by the Red Cross in an unconscious state with generalized tonic/clonic seizures. She had a history of epilepsy in childhood. Her mother reported that she had been raped and nearly strangulated prior to arrival. She was immediately intubated and placed on mechanical respiration. She was, however, assisting the respirator.

General physical examination showed the patient to be in a state of “akinetic mutism” with decorticate posturing. General physical examination was unremarkable except for evidence of lung consolidation consistent with pneumonia, with BP of 115/81, PR of 138/min, Temp of 38°C, and RR of 21/min.

Neurological examination showed the pupils to be 4 mm in diameter and only sluggishly reactive to light. Optic fundi were normal. Corneal reflex was preserved. Extraocular movements were present but sluggish. She was unresponsive to painful stimulation. There was generalized hyporeflexia. No Babinski's was detected. There was forensic evidence of attempted rape.

Laboratory tests included a chest X-ray which was consistent with pneumonia. She had a moderate leukocytosis. CPK was moderately elevated. The chemistry profile was otherwise unremarkable. An EEG was consistent with the clinical diagnosis of anoxic encephalopathy. CT and MRI of the brain were both normal.

*Course in the Hospital.* The patient was maintained on life support and conservative therapy. For seizure prevention she was started on phenytoin 100 mg tid. Throat and sputum culture grew methicillin resistant staphylococcus aureus (MRSA) requiring Vancomycin therapy.

A repeat neurological examination in the second week of hospitalization continued to show “akinetic mutism”. The patient now had spastic quadriplegia and she was experiencing decerebrate posturing. During the third week of hospitalization she developed incessant bruxism resulting in two broken teeth. A trial of clonazepam was unsuccessful in controlling the bruxism. A trial of bromocriptine also failed, although we elected to continue this drug in an effort to correct the patient's stuporous state by using this dopamine agonist. We then started the patient on baclofen 10 mg tid which in a matter of days corrected the bruxism. However, ten days later the patient had a recrudescence of the bruxism prompting an increase in the dosage of baclofen to 20 mg tid, this time with a sustained efficacy.

The patient required tracheostomy. However, her condition improved significantly in that she became responsive to simple commands and had no further posturing, although she was still quadriplegic. She was now afebrile. Throughout the hospital stay she received intensive physical therapy.

## 3. Discussion

Bruxism can occur during wakefulness or during sleep. Bruxism during daytime is commonly a semivoluntary “clenching” activity known as “awake bruxism” (AB) or diurnal bruxism (DB). AB can be associated with life stress caused by family-related issues or work pressure. Bruxism during sleep is called “sleep bruxism” (SB). SB is an oromandibular behavior that is defined as a stereotyped movement disorder occurring during sleep, characterized by tooth grinding and/or clenching [12]. Bruxism is considered to have multifactorial etiology. SB has been associated with peripheral factors such as tooth interference in dental occlusion, psychosocial influences such as stress or anxiety, and central or pathophysiological causes involving brain neurotransmitters or basal ganglia [13].

Other pathophysiological factors are also suggested to be involved in the precipitation of bruxism. Of note, the physiology of sleep has been extensively studied, especially with respect the “arousal response,” in an attempt to find the possible cause of this disorder. Arousal response is a sudden change in the depth of the sleep during which the individual either arrives in the lighter sleep stage or actually wakes up. Such a response is accompanied by gross body movements, increased heart rate, respiratory changes, and increased muscle activity. Lobbezoo et al. [14] in their study showed that 86% of bruxism episodes were associated with arousal response along with involuntary leg movements. This shows that bruxism is a part of the “arousal response”.

Recent theories point to the disturbances in the central neurotransmitter system may be involved in the etiology [14]. It is hypothesized that the direct and indirect pathways of the basal ganglion are disturbed in individuals with bruxism. The direct output pathway goes directly from the stratum to the thalamus whence afferent signals project to the cerebral cortex. The indirect pathway on the other hand passes by several other nuclei before reaching the thalamus. In case of bruxism there may be an imbalance in both of the pathways [15].

A short course of therapy with dopamine precursors like L-dopa inhibits bruxism activity [14] while chronic long term use of l-dopa results in increased bruxism activity [14]. Serotonin reuptake inhibitors (SSRIs) which exert an indirect influence on the dopaminergic system [16] may cause bruxism after long term use. Amphetamine [14] which increases the dopamine concentration by facilitating its release has been found to increase bruxism. Nicotine stimulates central dopaminergic activities which might explain the finding that cigarette smokers report bruxism two times more than the nonsmokers.

In our case we used a gamma-aminobutyric acid (GABA) agonist (baclofen) which may exert its effect on regulating neuronal excitability throughout the nervous system. In humans, GABA is also directly responsible for the regulation of muscle tone. GABA acts at inhibitory synapses in the brain by binding to specific transmembrane receptors in the plasma membrane of both pre- and postsynaptic structures. This binding causes the opening of ion channels to allow the flow of either negatively charged chloride ions into the cell or positively charged potassium ions out of the cell, resulting in hyperpolarization. GABA receptors have been identified electrophysiologically and pharmacologically in all regions of the brain [17]. Our data support the hypothesis that bruxism emanates from an imbalance in brain neurotransmitters. Specifically, the efficacy of baclofen (a GABA agonist) in our patient suggests involvement of GABA'ergic system in the pathogenesis of bruxism. We also propose further research into potential therapeutic modalities for this condition.
